# The bricolage mode of emergency medical teams in China: deficient and in urgent need of transformation—A qualitative study

**DOI:** 10.3389/fpubh.2024.1333820

**Published:** 2024-02-16

**Authors:** Li Wang, Ya-Wei Sheng, Xin-Ye Qi, Fang-shi Li, Xin-Yu Qiu, Shen Shao, Yue Du

**Affiliations:** ^1^School of Public Health, Tianjin Medical University, Tianjin, China; ^2^Public Health Emergency Research Center, Tianjin Medical University, Tianjin, China; ^3^The Quality Control Center of Basic Public Health Service, Tianjin Medical University, Tianjin, China

**Keywords:** emergency medical teams (EMTs), resource bricolage, full-time team, the grounded theory, emergency response

## Abstract

**Introduction:**

Emergency medical rescue plays a vital role in alleviating the harm of all kinds of emergencies to people's physical and mental health and life safety. The current emergency medical teams (EMTs) formation model is not unified. We focused on the disadvantages of the bricolage mode of China EMTs and put forward empirical-based countermeasures to improve the emergency management ability of EMTs.

**Methods:**

From March to September 2022, 23 leaders of EMTs in North China (Tianjin) were selected by objective sampling method to conduct one-to-half structured in-depth interviews. Nvivo12.0 software was used for three-level coding. The disadvantages of the bricolage model of EMT were analyzed.

**Results:**

Based on the three-level coding, 150 initial concepts, 36 sub-coding, 17 main coding, six categories, and two core categories were sorted out. Management structure, internal stability, and support are recognized as the crucial elements armed with the EMTs.

**Discussion:**

The bricolage EMTs have disadvantages such as a chaotic management structure, weak internal stability, and inadequate support. It is necessary to construct full-time EMTs that incorporate a standardized personnel admission mechanism, full-time training and exercise mechanism, diversified incentive mechanism, and multi-agent cooperation mechanism, etc.

## Background

World Health Organization emphasizes the importance of building Emergency Medical Teams (EMTs) to face the emergency and maintains that effective rescue plays an essential role in saving people's lives and economic and social development (1). However, responses to large-scale disasters, like those witnessed in Haiti and elsewhere, have varied significantly, ranging from well-prepared, timely, and life-saving responses to temporary, poorly-trained, ill-equipped, and even dangerous ones (2–4). In response to health threats. China has established national EMTs These teams are bricolage teams that consist of medical, nursing, logistics, pharmacy, and equipment departments, They are formed with limited resources and disbanded as per the requirements of different situations (5–7). Take the city of Tianjin in North China for example: EMTs adopt temporary bricolage methods, such as “human resources bricolage” and “material bricolage,” to build their teams (8, 9). The concept of “Human resources bricolage” involves assembling teams of people with different medical specialties and skills in medical institutions, giving the team members dual identities as both hospital medical staff and EMTs members. “Material bricolage” refers to pooling resources by combining internal resources within the medical institutions and seeking external social support.

This study used bricolage behaviors such as material and human bricolage to define the current mode of EMTs developed based on medical institutions. Bricolage refers to using available resources to re-deconstruct, recognize, or integrate existing resources to create new rules and accomplish tasks (10). It's a temporary and effective means for small or medium-sized enterprises and new start-ups to solve resource constraints problems. Bricolage EMTs undertake multiple tasks, such as daily clinical work, training requirements, and emergency medical rescue activities. However, due to the limitation in innovation capacity and lack of professional experience accumulation (11, 12), the temporary nature cannot provide fundamental solutions for teams' sustainable development and competence improvement. Moreover, clinical departments often refuse to send EMTs for training because of staff shortages and insufficient connection between training content and clinical work, which negatively affects the effectiveness of training exercises and the enthusiasm of team members to participate (13, 14). Therefore, improvised, poorly prepared, and under-equipped EMTs may undermine the effectiveness of emergency rescue (15). Thus, it is necessary to explore the existing challenges faced by EMTs and propose corresponding solutions.

To date, there have been few studies exploring the formation of EMTs, especially the problems associated with this “bricolage” approach. While many articles and studies have highlighted the crucial role played by EMTs in responding to various emergencies, there is little evidence to show that the way EMTs are formed has an impact on their effectiveness in emergency rescue operation. We conducted this study to better understand how bricolage behavior contributes to the deficiencies and limitations in the construction and development of EMTs, as well as how to mitigate these impacts. This study aims to provide alternative ideas and potential improvements for the WHO's EMTs formation Initiative and national EMT formation mode.

Grounded theory, a qualitative methodology that inductively generates theory, has been widely applied in the realm of emergency responses and management research. Khan et al. (16) followed a constructivist grounded theory approach explored the self-preparedness processes of frontline healthcare workers to deliver high-quality care during the COVID-19 pandemic. Li et al. (17) focused on the earthquake disaster experiences of Chinese nurses and developed an emergent theory using grounded theory and symbolic interactionism, which can aid in better preparing nurses for future disasters. So, in this study, we employed grounded theory as a theoretical framework to collect and analyze data derived from in-depth semi-structured interviews. The primary objective was to provide empirical evidence to promote the establishment of full-time and standardized management of EMTs, allowing them to fully utilize their skills and expertise in emergency situations.

## Methods

### Study design

This study includes two main parts. The first part comprises a cross-section questionnaire survey of the EMTs in Tianjin, a municipality directly under the central Government of China. The basic information table mainly focuses on the following contents: (i) Subsidiary units, level, category, and number. (ii) Annual working funds of the team, sources, and uses of funds; (iii) Team training frequencies, ways, and terms. The second part is the interview survey about the operation of EMTs. The main contents of the interview based on the literature are as follows: (i) Please introduce the detailed information to your EMTs and the ways obtained, including the existing human, material, and financial resources; (ii) The integrate means of your EMTs regarding human, financial and material resources; (iii) Please introduce of the management mode to your EMTs, such as personnel access mechanism, assessment and incentive, training and drill mechanism, etc.; (iv) How about the working atmosphere, trust level, team cohesion and cooperation level among the team members; (v) In your opinion, which aspects need to be improved or enhanced to improve the overall performance of the EMT? Specific items are in Appendices 1, 2.

### Participants

From March to September 2022, 23 EMTs' leaders, who were also EMTs members, were selected by objective sampling to conduct one-to-one semi-structured interviews. The inclusion criteria of participants were as follows: (i) had participated in on-site emergency medical rescue work; (ii) had the intermediate title or above, bachelor's degree or above; (iii) had more than 10 years of working experience related to emergency medical rescue work; and (iv) had a high interest in this study and can provide objective and comprehensive opinions. Participants who self-reported not having been involved in field rescue were excluded from this study. The demographics of the interviewees are shown in Table 1. We have allocated 800 RMB from the scientific research fund to each participant as a token of appreciation for their interview.

**Table 1:** Characteristics of participants interviewed (*N* = 23).

**Demographic variables**		**Frequency (*n*)**	**Percent (%)**
Gender	Female	6	26.1
	Male	17	73.9
Age	≤ 40	8	34.8
	41–50	11	47.8
	≥51	4	17.4
Education	Bachelor's degree	7	30.4
	Master's degree	9	39.1
	Doctoral degree	7	30.4
Experience in emergency medical rescue	≤ 15	7	30.4
	16–20	7	30.4
	21–25	3	13.0
	26–30	5	21.7
	≥31	1	4.3
Research field	Health emergency management	5	21.7
	Emergency medicine	2	8.7
	Psychological crisis intervention	2	8.7
	Breathing epidemiology	3	13.0
	Others	11	47.8

Based on strong medical and health institutions and affiliated hospitals of the Armed Police Logistics College, two national EMTs and five municipal EMTs of five categories have been established in Tianjin. Among them, the China International EMT (Tianjin) was built based on the National EMT established by Tianjin People's Hospital. It passed the mid-term certification of the International Medical Emergency Team (Category 2) of the World Health Organization on April 26, 2019, becoming the fourth international EMT in China and the 24th in the world. The Armed Police Special Medical Center has built China's first rescue medicine training base and advanced digital Mobile Cabin hospital. It has successfully completed many major national and military medical support tasks and has been named “National EMT” and “National Psychological EMT.” The other five provincial-level EMTs are Tianjin Fourth Central Hospital, Tianjin Nuclear and Radiation Emergency Response Team, Tianjin Anding Hospital, Tianjin Disease Control and Prevention Center, and Tianjin Occupational Disease Prevention Hospital Chemical Poisoning Emergency Backup Rescue Team. The team categories are emergency medical rescue, nuclear radiation, psychological crisis intervention, infectious diseases, and chemical poisoning. The basic information (team names, levels, categories, number of people, annual working expenses, etc.) and training (training methods, content, etc.) of Tianjin EMTs are in Appendices 3, 4.

### Data collection and quality control

Based on the previous research on optimizing the talent selection mode of three EMTs in Tianjin, the grounded theory was used to discuss the composition mode and team construction of China's EMTs. The study was approved by the Ethics Committee of Tianjin Medical University (TMUhMEC2020024), and verbal informed consent was obtained from the participants before the interview. The research team conducted one-on-one interviews both offline and online. Offline interviews were conducted in person in the interviewee's hospital office or conference room, and online interviews were conducted using the TenCent Meeting platform. Data were collected through semi-structured personal interviews and basic information questionnaires. All interviewees were trained in advance to reduce the bias and Hawthorne effect in the interviews, mainly focusing on raising questions, listening, and feedback. Before the interview, the research team learned about the interviewee's background information, such as personal information and rescue experience of the interviewees by consulting the hospital's official WeChat account or official website. The interview moderator introduced himself and explained the purpose of the research to arouse the interviewee's interest in this study and obtain more information. Then the EMT leader gives a general description of the rescue team and fills out the form. The average interview duration was about 1–2 h, and the duration was gradually shortened due to theoretical saturation.

During the interview, audio was recorded with the consent of the interviewees. We used the iFlytek sound recorder for live recording. Written notes were also made during the interview as a backup to the audio recording and to follow up on important points discussed earlier in the interview. Interview transcripts were converted by cross-checking the audio files with word-for-word accuracy. Two people carefully reviewed and compared the transcript against the audio files, and finally transcribed the written interview notes into Microsoft Office Word (Seattle, WA, USA) for encoding.

### Data analysis

This study used the grounded theory method to conduct the exploratory analysis of interview data and conducted coding work while collecting data (18). Grounded theory is a research methodology based on data, and uses a bottom-up research logic. It requires systematically collecting and analyzing empirical data regarding particular phenomena, gradually conceptualizing and categorizing, and finally discovering, developing, and testing the theory from the data. Open coding analysis follows three steps: labeling, conceptualization, and categorization. Axis coding is to discover and establish the links between the main categories and their subcategories and to explore the interrelationships between various categories. Selective coding is to explore the core category under the discovered category (19–21).

The research aimed to identify the disadvantages of EMTs, and develop a feasible path to promote the development of bricolage EMTs within the context of interpreting these concepts and frameworks. The research team considered the suspension of presuppositions and hypotheses, compared the research data word by word, thoroughly explored the initial concepts and categories, and named them using the local concepts of the respondents.

**Figure 1:**
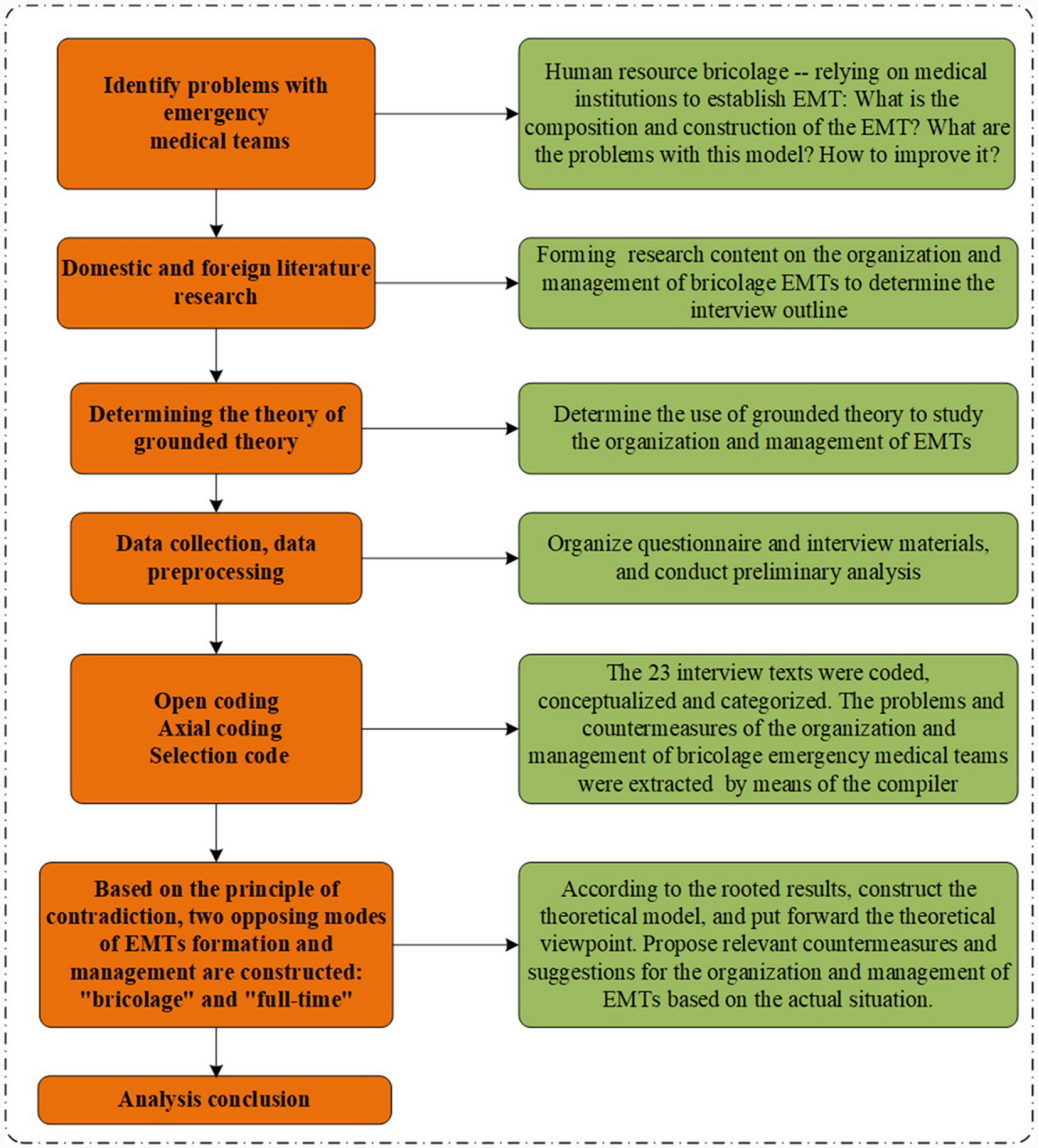
Flow chart of grounded theory research.

SPSS20.0 software was used for general descriptive statistical analysis of the basic information of interviewees. Nvivo12.0 software was used to mark interview data word-for-word, record memos, extract sentence frequencies, and conduct substantive third-level coding according to the grounded theory research steps to test the theoretical saturation. Nvivo12.0 software is only used to assist in coding classification, and the coding work is completely completed by the members of the research group. The detailed flow chart is shown in Figure 1.

To ensure reliability and validity, two researchers participated in the coding process, and any differences between the researchers were resolved through group discussions and literature reviews to attain internal consistency within the coded data. Data collection and analysis reached theoretical saturation at the 23rd participant, with no new concepts, categories, and relationships found, indicating that the theoretical model passed the theoretical saturation test.

## Result

In this study, we analyzed the interview data and classified the resources to understand the resource situation of the rescue team. Table 2 shows the resource status of the bricolage EMTs. Due to the scarcity of resources, EMTs in North China predominately rely on internal and external resource acquisition efforts; the former includes in-house hospital supplies, while the latter includes government funding, social donations, and self-financing. Human resources were the foundation of effective EMTs and supported the operation of the whole rescue team. The personnel specialization gives team members more time and resources to participate in training and drills to improve their knowledge and skills. Adequate financial resources acted as the necessary backing for equipment purchase, personnel subsidies, operation and maintenance, training, drills, etc. Adequate material and human resources are used together to optimize the completion of rescue tasks.

**Table 2:** Resource status of bricolage EMTs of this study (*N* = 23).

**Resource dimension**		**Original statement**	**Frequency**
Personnel	Sufficient	The personnel are sufficient, because we rely on hospitals	5
	Insufficient	It is difficult to assemble an emergency medical rescue team in the first place, and more personnel were required in emergency	18
Work form	Part-time	I may wear several hats by myself. I'm not only a member of the rescue team, but also have my own full-time job in the hospital	23
	Full-time	None	0
Money	Sufficient	None	0
	Insufficient	As a bricolage emergency medical rescue team, our team is facing financial difficulties, involving equipment, training funds, and training	23
Material	Sufficient	We have complete equipment such as ventilators and rescue equipment, because we are a rescue training base	3
	Insufficient	Protective suits are reused, and supplies are scarce at first	20
Resource bricolage model	Using internal resources	We try to integrate our own resources and use the existing conditions to complete the task	10
	Seeking external support	We negotiate and communicate with some friendly neighboring units, governments, and scientific research institutions to obtain medicine, medicinal equipment, and other resources	13

### Open coding

A total of 150 initial concepts were formed in this study. After continuous comparison of these codes and the raw data considering similarities and differences, the repetitive concepts were combined into 36 sub-categories, including work maladaptation, high staff turnover rate, vague assessment criteria, job attraction, low recognition, policy support, media publicity, and team atmosphere, etc.

### Axial coding

**Figure 2:**
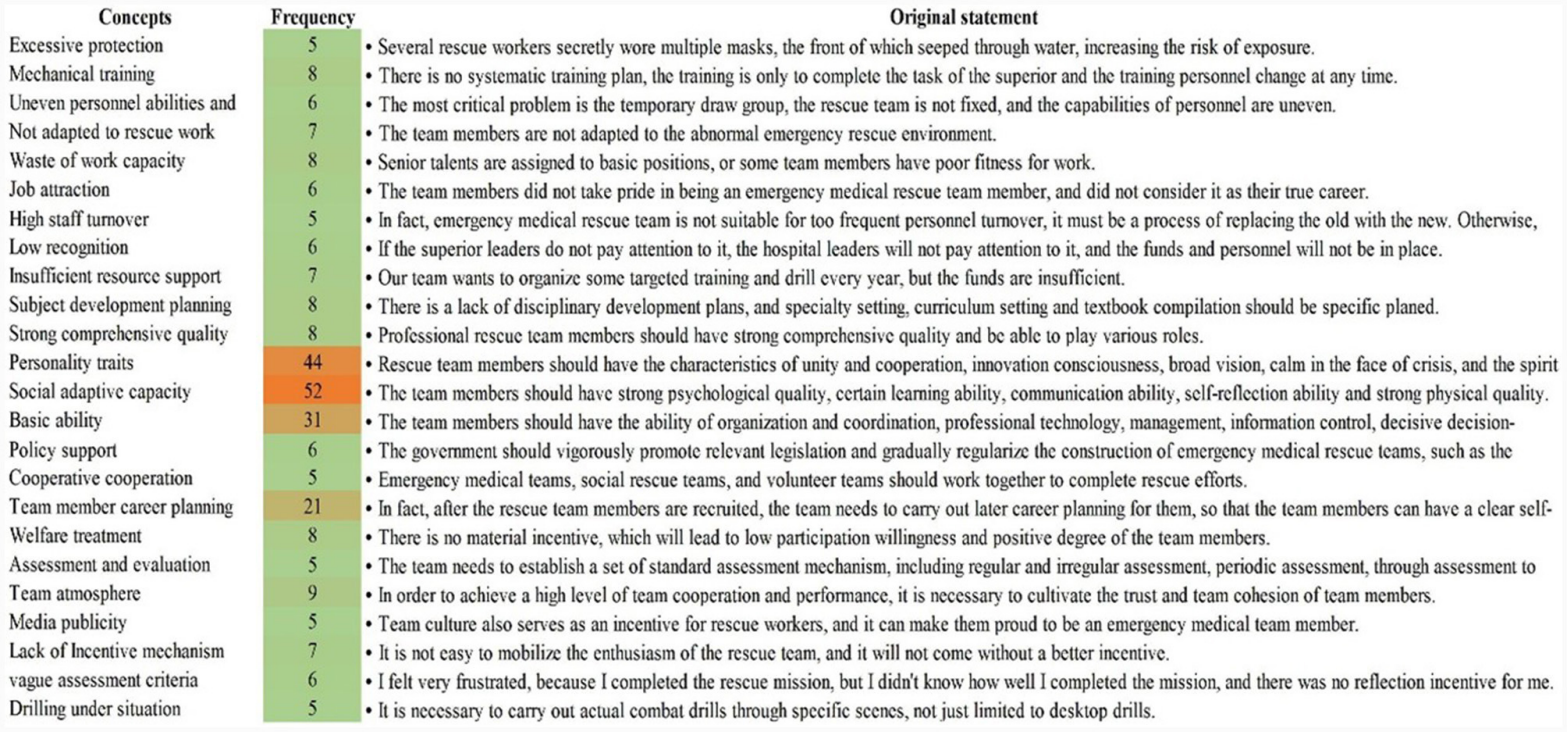
A subcategory formed after open coding.

By further exploring the logical relations between the sub-categories under open coding and integration, 150 initial concepts were summarized in 17 subthemes and six themes (see Figure 2 and Table 3).

**Table 3:** Axial and selective coding results.

**Paradigm**	**Category**	**Main categories**	**Deputy category**
Difficulties and drawback (bricolage EMTs)	Chaotic team management structure	Lack of regular training and drills	Mechanical training exercises
			Weak awareness of protection
		Unscientific personnel selection and staffing mechanism	Not adapted to rescue work
			Waste of work capacity
		Preplan management confusion	A preplan with a single form
			Preplan update delay
		Non-standard equipment management	Waste of resources (idle equipment)
			Environmental applicability of equipment
	Weak team internal stability Poor team support	Low team cohesion	Job attraction
			High staff turnover
		Lack of assessment and incentive mechanism	Lack of incentive mechanism
			Vague assessment criteria
		Lack of leadership attention	Low recognition
		Insufficient resource support	Lack of resources
Action and strategy (full-time EMTs)	Improving the team management level	Talents cultivating system	Subject development planning
		Regular training and exercise mechanism	Training under situation construction
			Stress training and preplan training
			Popular science knowledge, continuing education
			Comprehensive exercise of multiple departments
		Scientific personnel selection	Strong comprehensive quality
			Social adaptive capacity
			Value concept
			Personality characteristics
		Standardized management of preplan	Diversification of preplan preparation
			Personnel rapid response system
			Logistic support system
		Establishing a medical equipment system	Resource modular allocation
			Developing convenient equipment
	Increasing team internal stability	Establishing incentive mechanism	Job attractiveness
			Target motivation
		Enhancing team cohesion	Team atmosphere
			Media publicity
	Increasing team support	Leadership attaches high importance	Policy support
			Ensuring the investment of funds
		Establishing a coordination mechanism	Cooperative cooperation mechanism
			Adding combat missions

#### Chaotic team management structure

The team management structure is considered an important factor in effectively managing pharmaceuticals and consumable medical equipment supply chains during disasters. This central theme contains four subthemes: training and drill, staffing, preplanning, and equipment management.

- Lack of regular training and drills

The aim of the training is to develop and cultivate the emergency rescue ability of the team members. Several interviewees indicated that there was no systematic training and that the existing training needed to be more extensive. Additionally, there appears to be inconsistency among the individuals receiving the training, as they change from time to time. Furthermore, the scheduling of their training is often determined by the changes in hospital schedules, rather than being primarily focused on the training itself for its own sake.

About the training and drill, one of the participants declared that:

“One of the most important reasons why people are not trained effectively is that today you received training, but you don't know who will conduct the training next year. Training activities do take place annually, but less and less.” (Participant 22)

Another participant added:

“Many operations do not wear gloves. From the overall point of view, the key is to raise awareness, especially self-protection awareness.” (Participant 20)

- Unscientific personnel selection and employment mechanism

In terms of personnel, this study found that personnel representatives are responsible for conducting human resource selection, allocation, and quality assessment for emergency medical rescue personnel. Several participants said that emergency medical responders were not selected scientifically, and the utilization rate of the team was low. Unscientific admission process contributes to considerable disparities in team members' skill levels, and a low utilization rate will result in a decline in the overall skill level of the team. Thus, team members may need time to adjust to their roles within the emergency medical teams (EMTs). However, this transitional period can impact their ability to perform their regular hospital duties effectively, such as hospital routine diagnosis and treatment of patients.

A participant said:

“If it is only activated in the event of a major medical disaster, the daily presence of the team will be too low, and it will be challenging to build the team firmly. In fact, the rescuers have never experienced this kind of environment before and may be confused. Besides, many members will not adapt to the abnormal rescue.” (Participant 8)

- Preplanning management confusion

Preplanning management, as another subtheme, was mentioned here. Preplanning management is a preventive preparation to allocate human resources and deal with emergencies. Participant 6 implied the importance of preplanning management during EMRs. Some participants pointed out that the rescue EMTs' plans have not changed for years and use only one set of plans.

In this regard, one of the participants stated:

“Your plan may only be about the earthquake or the fire, but the various emergency scenarios may involve earthquake plus fire. You can't just use a single one to face the uncertain situation.” (Participant 5)

- Non-standard equipment management

Finally, as the material resource support for emergency rescue, equipment management was one of the essential subthemes. This subtheme is becoming an increasingly crucial aspect of emergency rescue, given that the smooth development of rescue activities depends on the availability of emergency resources and equipment. Some interviewees indicated that certain pieces of equipment may become unusable and need to be scrapped if left unused for an extended period due to their limited shelf life, and material support was not uniformly planned.

One participant remembered his experiences as follows:

“Many things have the expiration date, especially food. If you placed one year or two, it will be expired. If you don't use stored materials, it will be scrapped. The investment needs in equipment and supplies are huge.” (Participant 10)

#### Weak team internal stability

The internal stability of EMTs means team members willing to stay in EMTs. The related subthemes in this regard were: human assessment and incentive drive mechanism and team culture construct to maintain human resources.

- Low team cohesion

Team cohesion measures how well team members work with one another. A cohesive team is characterized by a shared understanding of roles, an embracing of individual strengths, and a commitment to overall team goals. However, several participants indicated that most team members did not regard their work on the emergency medical team as a real profession. This highlights the need for a stronger sense of professional identity within the emergency medical team, particularly in the light of the overly frequent personnel turnover.

One participant declared that:

“From the perspective of team members and leaders, they don't seem to take emergency rescue work seriously as a real daily job and think this is not a serious matter. Thus, there is a certain degree of autonomy and mobility of personnel. In fact, the orderly work of the emergency medical team itself is a process of replacing the old with the new, while it is not suitable for emergency personnel turnover too often, or the old tradition and experience will be lost.” (Participant 14)

- Lack of assessment and incentive mechanism

According to the present results, the lack of assessment and incentive mechanisms was an obvious problem in EMTs. Most participants said that emergency medical rescue work does not have clear standards regarding material and spiritual incentives, such as personnel subsidies, which makes it difficult to motivate team members Furthermore, there is a lack of proper assessment, feedback, and summarization of the team members' performance in completing the rescue task.

Two participants clarified as follows:

“Can the rescue work command leaders tell me the specific criteria when selecting personnel or assigning tasks? More often, they only told me the task, but no standards, checks, or evaluations, which frustrated me. And I didn't know if the work that I had done was good or not.” (Participant 3)

“I went out to participate in the training exercise, but I had to continue to work in the hospital after returning. The consequence is I received less money when the hospital sent out the performance at the end of the year, while the rescue team did not have corresponding subsidies. Without a certain moral and material motivation, people will not recognize the rescue team's work.” (Participant 23)

#### Poor team support

During emergencies, the need for team support becomes evident. It is a cost-effective and highly customizable solution for managing customer service tickets and coordinating team efforts. The related subthemes were leadership attention and sufficient resource support. The support strength of the emergency medical rescue team can be divided into two main aspects: internal support (hospital leadership support) and external support (directly affiliated unit leadership and government). Internal support from hospital leaders is demonstrated through the provision of relatively flexible training schedules. External support includes the investment of financial and various material resources, as well as facilitating communication and collaboration between provincial or inter-regional teams.

- Lack of leadership attention

Most participants indicated that both superior leaders and hospital leaders ignored emergency medical rescue work and were unwilling to invest substantial funds toward it, with the belief that it would not yield any tangible returns.

In this regard, one of the participants clarified his experience:

“No leader participated in our team at ordinary times. The superior and hospital leadership don't pay attention to the team's development, the funds are unavailable, and the personnel is incomplete. For example, I want to organize a drill in May, but each team member is under each department director or head nurse's management. You must make a report to the hospital leader, while the hospital leader said that work has been busy recently, and it is not a good time to drill. However, rescheduling may mean the drill does not exist.” (Participant 13)

- Insufficient resource support

Adequate resources encompass both staffing and equipment necessary to provide effective responses that meet the requirements of the different emergency situations. Most interviewees said that if we want the team to continue developing and improving relevant capabilities, the current support of funds and various material resources falls short of meeting such objectives.

One participant declared:

“Our team is short of money and resources. For example, we need necessary auxiliary facilities for psychological crisis intervention, including software and tools. Besides, we don't have the funds for scale training.” (Participant 22)

#### Improving the team management level

By adopting a revised establishment mode, scientific management of EMTs can be achieved. Key measures include establishing a comprehensive personnel training system, implementing regular full-time training and exercises, setting clear personnel selection criteria, ensuring a well-equipped medical equipment system, and implementing standardized preplanning management of EMTs.

- Talents cultivating system

Well-defined discipline development planning is key to cultivating and reserving talents for EMTs. Some participants believed that the development of EMTs lacks a proper discipline development plan, resulting in the absence of necessary disciplinary frameworks and corresponding curricula that cover areas such as management, interpersonal communication, ethics, and professional skills.

One participant declared:

“There is a lack of discipline development planning. How many emergency medical rescue specialties should be established needs discussion. In fact, there is a big cause, which is that every major disaster will be followed by a major epidemic, but this is something that has not been appreciated.” (Participant 5)

- Regular training and drill mechanism

Regular training and drill mechanism was identified as one of the important subthemes. Such a mechanism allows trainees to receive training repeatedly to master new information and skills. It also places emphasis on drill exercises, helping trainees attain proficiency in practical skills like performing new acts through repetitive exercise. These training and drill exercises reward trainees with immediate feedback, further enhancing their overall performance.

Two participants stated:

“The drill should be scenario-built and closer to actual combat. Instead of a single team, multiple teams should be added to the drill. We should do the joint drill.” (Participant 7)

“Because early mental construction is not enough, maybe when you work for a while, you miss home, you can't stand it. This kind of thing is really early training. When you are lonely and can't see your family for a long time, how to reduce stress and counseling is also needed.” (Participant 5)

- Scientific personnel selection

Scientific personnel selection is emphasized as another significant subtheme. Some experts suggest that it is necessary to recruit compound rescue talents with strong comprehensive qualities. Ideal rescue team members need to have specific social adaptability traits, such as resilience, self-reflection, and adaptability; certain values concepts, such as perseverance and fearless dedication; and personality characteristics, such as unity and cooperation.

One participant noted:

“When choosing team members, managers should choose people with comprehensive solid quality.” (Participant 8)

- Standardized management of preplanning

Most participants have indicated that emergency medical teams need to develop diversified preparedness lists tailored to different types of disasters. The content of the plan should include personnel rapid response, logistics management, and other essential mechanisms.

One participant added:

“As for the whole process of our tasks, we need to know who will distribute the tasks, who will obtain the tasks, who will coordinate with each other, and who will guarantee the tasks. In terms of mechanism and system, we need to have a complete pre-plan mechanism.” (Participant 3)

- Establishing a medical equipment system

Some interviewees said that equipment should be modular to reduce resource waste. They also stress the need for the development of more portable equipment to alleviate the burden during emergency response.

In this regard, one of the participants emphasized the establishing of a medical equipment system as follows:

“In view of team building, it is necessary to carry out modular allocation of health resources. For example, in the “3+X” or “4+X” mode of college entrance examination and high school entrance examination, the basic ability is 3 or 4, and the latter “X” is configured according to different situations to play different roles for different things.” (Participant 21)

#### Increasing team internal stability

The present results have emphasized the importance of increasing internal stability within EMTs. The implementation of a full-time construction mode can advance the establishment of a personnel assessment and incentive mechanism for EMTs, thereby fostering stronger team cohesion. The main subthemes here are as follows: incentive mechanism and team cohesion.

- Establishing incentive mechanism

The incentive and assessment mechanism mainly includes job attraction and goal incentives. According to the participants, emergency medical rescue teams need to develop career planning for members, provide spiritual feedback to members through setting good examples and media publicity, and increase welfare benefits to enhance job appeal. Moreover, the team evaluation mechanism should be established to help the team members in self-positioning to achieve the goal of motivation.

One participant exemplified:

“There should be an institutional incentive for rescue teams to volunteer. And then, there will be some personal spiritual growth while performing the task, which will bring high-level feedback to everyone. At the same time, some material rewards are also necessary.” (Participant 3)

- Enhancing team cohesion

The participants have mentioned team cohesion as a determinant in the success of emergency rescue activities. Managers can enhance team cohesion by creating an atmosphere of collaboration and unity, as well as through cultural promotion. For example, emphasizing respect for the emergency medical rescue personnel, instilling pride in being an emergency medical rescue team member, and nurturing a deep commitment to the cause of rescue.

A participant afflicted in a military organization clarified:

“Because the first young man is not looking for money, but he feels honored to be in the team. Then he can learn something that will help him develop in the future. In addition, the whole staff has formed respect for the rescue workers, and everyone is proud to be in the rescue team.” (Participant 23)

#### Increasing team support

Given the basic conditions of forming a full-time EMT, the policy and resource support will be enhanced, and the rescue cause will draw attention from leaders. EMT will transition into a rewarding career path. According to the participants, team support was divided into leadership attention and coordination mechanisms.

- Leadership attaches high importance

The smooth execution of all tasks depends on the leader's attention to the emergency medical rescue. The improvement of leaders' attention to emergency medical rescue is mainly manifested in increasing fund input, provision of policy support for personnel selection, and the establishment of training and drill mechanisms.

One of the participants emphasized on the leadership attention as follows:

“Without government support, public health cannot continue. It is what the government should do, not a hospital to do, let alone a department or a person can do. The piece of legislation is the most important. This system includes personnel incentive, personnel team management and base construction, and other aspects.” (Participant 21 and Participant 22)

- Establishing a coordination mechanism

And finally, time-wasting was identified as another crucial subthemes. Some participants suggested building small, focused emergency medical teams that integrate search-and-rescue medicine. As multiple agencies are typically involved in the rescue process, the coordination can be challenging and complex. Unlike the straightforward coordination within a system or hospital, it may require cross-unit, cross-department, or even a series of cross-professional collaborations. Therefore, it is crucial to establish improved mechanisms for coordinating and integrating these various entities.

In this regard, a participant declared that:

“We should integrate the characteristics of rescue teams in hospitals and rescue organizations in society, strengthen communication, and focus on integration. At the same time, the emergency medical rescue team should enhance communication with scientific research institutes and enterprises because enterprises have good research and development capabilities.” (Participant 20)

### Selection coding

**Figure 3:**
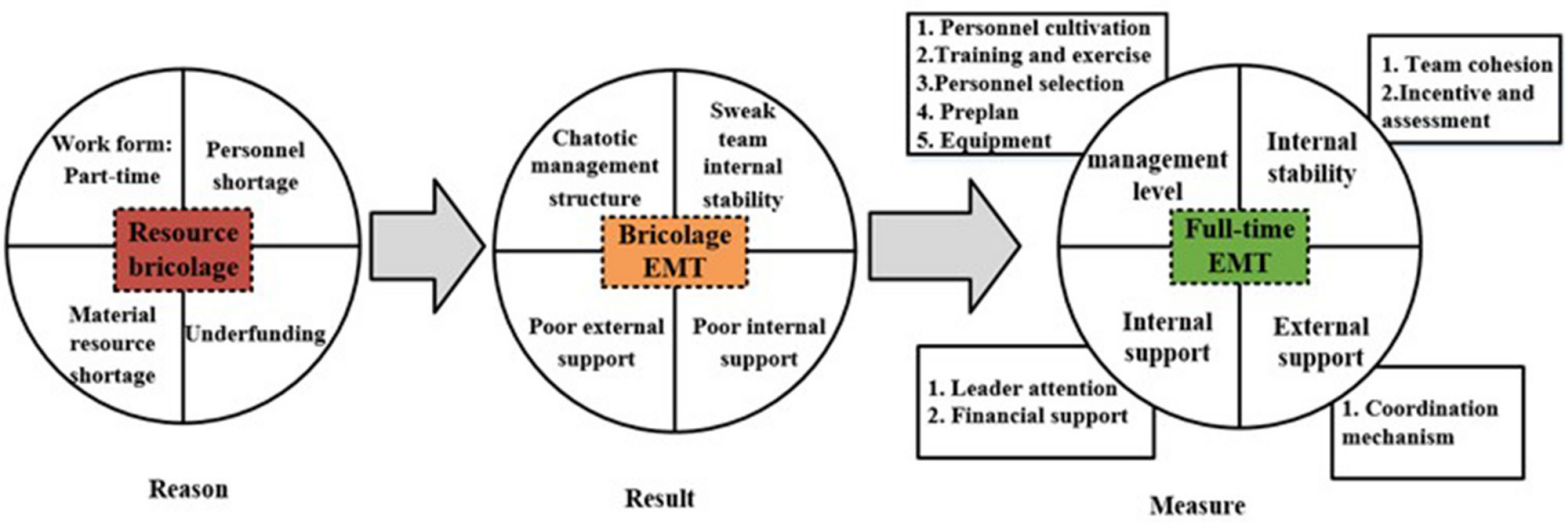
Theoretical model of improving emergency management ability of full-time EMTs.

The most important core categories in the study are identified by summarizing the secondary categories generated by the axial coding. The formed core categories can be connected into a complete narrative: in the bricolage EMTs in North China, the team management structure is chaotic due to the low awareness of protection, unscientific staffing allocation, and the lack of regular training and drills. Low team cohesion and lack of assessment and incentive mechanisms led to weak internal stability of the team; Low management recognition and inadequate resource support led to poor team support. Therefore, it is necessary to make EMT management more scientific, improve internal stability, and enhance support. This can be achieved through the implementation of a standardized personnel access mechanism, full-time training and drill mechanism, diversified incentive mechanism, multi-agent cooperation mechanism, and increased attention from leaders. Figure 3 shows the theoretical model.

## Discussion

The WHO EMTs initiative calls on countries to establish national EMTs to respond to excessive healthcare needs in their countries due to emergencies or impairment of existing capacities and to reduce disability and death (22, 23). China has set up 40 national-level EMTs (24), involved with comprehensive rescue, poisoning treatment, and nuclear radiation treatment (see Appendix 5). The emergency management ability of full-time EMTs was the main determinant theme of the study. During a crisis, emergency rescue work can encounter various problems in the team management structure, internal stability, and support. The bricolage EMTs could be more disorderly, with an incomplete organizational structure, a strong tendency to be arbitrary in allocating and adjusting health human resources, and a lack of personnel admission mechanisms (25, 26). This study aimed to develop a theoretical model for improving the emergency management ability of full-time EMTs.

### Bricolage EMTs: chaotic team management structure

Notably, Canada's Disaster Assistance Response Team (DART) conducts strict qualification reviews and certification for its members. Candidates need to pass professional knowledge and psychological resilience evaluations to be included in the rescue system (27). Similarly, the United States and Japan have integrated disaster mental health services and emergency management into basic education and established emergency training courses (28, 29). A third-party management organization has been established to evaluate recruitment and assess the ability of rescue team members in the UK (30). However, China has not established an assessment and evaluation mechanism, because China's EMT is only set up temporarily to deal with emergencies, and it does not have a complete mechanism. Moreover, the reward and punishment mechanism cannot be implemented after the assessment, because the team members do not receive any subsidies or salaries, and it is the greatest effort to mobilize personnel to participate in emergency medical rescue work (31). Professional personnel selection standards are the insurance of high-quality emergency rescue tasks. Without them, achieving ideal rescue outcomes becomes difficult. Therefore, China's EMTs need to establish a flexible registration and certification mechanism for rapid response emergency medical technicians and strictly limit the entry threshold through professional qualification certification to select competent members for the EMTs (32).

Additionally, the United States has developed a special national training plan regarding classroom, field, commanders, and teams for National Disaster Medical Assistance Teams (DMATs) (33). Singapore has established local and foreign courses for EMTs' members to gain a comprehensive understanding of emergency rescue systems worldwide (34, 35). Lei et al. mentioned that the current rescue system in China suffers from dispersed forces, leading to a lack of standardized continuing education and training mechanisms. The majority of personnel have not received rescue training, resulting in inadequate professional skills needed to meet the needs of rescue work (26). There are two main factors contributing to this issue. First, members of EMTs have taken on multiple roles and have very limited time to actively engage in emergency rescue work due to the routine diagnosis and the high intensity of clinical diagnosis and treatment work in hospitals (36). Thus, training and exercise seemed merely formalities in China. Secondly, the lack of emergency rescue experience and systematic training limited the ability to adapt to rescue work in various complex disaster situations and the effectiveness of on-site treatment (37, 38). Therefore, it is necessary to establish a regular management mechanism for medical institutions to ensure sufficient time and systematic training in China. One possible solution is to develop an online platform where members of EMTs and other stakeholders can access and share knowledge, theories, and skills related to emergency medical rescue. In addition, joint training and exercises should also be conducted at national and cross-regional regional levels.

Besides, the interviews of this study also revealed that the bricolage EMTs have not updated the plans in sync with specific scenarios or the latest advancements in information technology, and there is a lack of contingency plans suitable for different scenarios based on practical experience and actual rescue conditions. Japan has emphasized prevention and rational self-rescue as priorities in emergency medical rescue disaster response guidelines based on the derived situations of disaster categories formulated. Given the hazards of delay in emergency response work, it is necessary for China to develop different plans for different types of disasters and update the existing plans according to environmental changes to make emergency medical rescue plans more scientific and applicable (26).

### Bricolage EMTs: weak internal stability of the team

A low sense of identity, a high turnover rate, and a lack of standardized assessment and incentive mechanisms are all indicators of weak internal stability of the bricolage EMTs. Scholar Aitken said that because the team members are temporarily transferred from various departments, they often have temporary ideas, leading to poor team stability and difficulty in implementing performance management (39). Weiner et al. (40) argued that the U.S. Disaster Medical Rescue Team is led by a core of trained professionals from all fields, and they can be immediately deployed in challenging and stressful situations. In China, the temporary and bricolage nature of EMTs has led to poor internal stability and low cohesion On one hand, temporary mobilization and part-time status of team members make the EMTs lack cohesion and attractiveness. Rescue team members are all part-time workers, and the lack of clear career development paths within the team makes it difficult for them to view emergency medical rescue work as their real career. Additionally, the random pairing of personnel from different departments has led to problems such as non-uniform team structure, uneven personnel quality and abilities, confusing team member role recognition and low team identification, and high turnover rates (41), ultimately leading to weak internal stability within the team.

Besides, the temporary bricolage work mode also makes performance management difficult to implement effectively. At present, the incentive mechanism of China's temporary teams is not mature, and there is a lack of means such as salary and promotion of professional titles to incentivize team members. Additionally, assessment standards for members' ability, participation in training and exercise tasks, and completion of rescue missions are unclear and not linked to performance rewards and punishments, with poor filing of assessment records further exacerbating team instability (42).

In contrast, full-time EMTs with specialized personnel lead to more standardized post-settings and an extended team survival time. The United States has established a team member assessment file to regularly evaluate and record their dynamic ranking in the same group. In this case, the personnel lists are updated based on the annual assessment results, and the rescue results are tracked and fed back for rewards and punishments (43). Therefore, salary, welfare, and employee incentives were the priorities of participators in rescue missions (27). Firstly, points are allocated and accumulated within the scoring period according to the different levels, ways, and participation in the training or drill. Top-ranking team members within the scoring period should be rewarded, while those who are at the bottom should be eliminated. Team members should be motivated through a combination of spiritual and material rewards, such as being awarded outstanding team member awards, being included in the core group of team management, and being awarded commemorative medals. Secondly, clear career plans need to be developed for team members to help enhance their sense of identity, clarify their responsibilities, tap their potential as rescue team members, realize self-growth, and enhance the attractiveness of EMT posts through material and spiritual incentives. Thirdly, according to Mayo's “social man” hypothesis, social behaviors such as social interaction and group belonging can improve the enthusiasm of team members (44). A good social relationship should be established between the rescue team leader and the rescue team members as well as among other team members. The team leader can create a collective atmosphere and team culture by organizing seminars and exchange meetings. Adopting democratic management principles can help establish effective communication channels and show respect for the participation and suggestion rights of the team members. New media platforms can be useful in promoting team culture and cultivating a sense of collective honor and cohesion within the team (45).

### Bricolage EMTs: inadequate support mechanism

This study also found that the support from government and hospital leaders and other subjects for the bricolage team is weak. Firstly, in terms of policy support, the management mechanism and fund investment mechanism of the team need to be further standardized in relevant legislation, and the existing funds cannot fully meet the needs of the team in equipment procurement, personnel subsidies, operation, and maintenance, training, and drills. As scholar Sun mentioned, the future success of the emergency medical team response depends not only on the compliance and change management of EMTs but also, to a large extent, on strengthening national disaster management arrangements and disaster laws in affected countries (41). Secondly, Leaders in medical institutions do not give enough support in terms of time and resources to EMTs that rely on hospitals for development. The training and exercise time of team members needs to be uniformly scheduled and arranged by the hospital leaders. However, these leaders often prioritize the completion of hospital diagnosis and treatment tasks for team members instead of allocating time for training and exercises. Additionally, the hospital has not yet formulated corresponding policies to support the sustainable development of the team, and the emergency supplies reserve still needs to meet the requirements of the rescue team (43). Thirdly, emergency rescue work entails a substantial workload and strong interdependence, often requiring the participation of multiple departments or collaboration across regions. This usually necessitates the centralized coordination of resources by the government and the establishment of an information-sharing platform. However, the bricolage team has limited access to various information and resources, and the recognition of their roles is insufficient, hindering their ability to achieve optimal rescue effects.

Management can supplement resources for the team and promote the development of EMTs by providing strong policy support. Japan has made national efforts to build a disaster relief system and strengthen the normalization of the emergency management system (29). Germany has a relatively comprehensive legal framework for disaster emergency response, and its legal system mainly includes the Basic Law of the Federal Republic of Germany, the Law on Civil Protection and Disaster Relief, and relevant provisions of various states (46). Therefore, the Chinese government needs to increase investment in personnel, equipment, and operation, and maintenance of EMTs, improve the support measures, and address the shortage of human, material, and financial resources of the teams. Also, the legal system needs to be improved, and the management mechanism, organizational support, and rights and obligations levels of the team need to be standardized to provide the team with greater benefits and autonomy in carrying out rescue tasks (37). The Singaporean government has established an “emergency response team” and a relatively complete emergency medical rescue system. Civil defense forces are responsible for fire-fighting and rescue activities, early medical treatment, and evacuation during disasters. The Ministry of Health is responsible for medical and public health testing, epidemic prevention, and triage, including taking over on-site treatment by civil defense forces, evacuating the wounded to hospitals, decontaminating contaminated personnel, determining casualties, and comforting the families of the injured. The police force handles the deceased and determines their identities (35). Therefore, the development of China's EMT needs to win support from various government departments, enterprises, institutions, and the whole society through a multi-departmental cooperation mechanism. Moreover, regional communication and collaboration should be strengthened to continuously promote the sound development of the team, facilitate knowledge and information sharing, and constantly improve the emergency response capabilities of the team (47). In the context of various compound disasters, it is difficult for bricolage EMTs to perform a high-quality emergency rescue operation. Establishing full-time EMTs can better enable the completion of regional and even trans-regional rescue tasks.

Establishing full-time EMTs is the current demand of China's bricolage EMTs. According to the interview data, the team leaders strongly hope to establish a full-time EMT in the future, which is mentioned as many as 12 times. The sample interview materials extracted are expressed as follows: “If you want to do this thing (emergency medical rescue), you must have a full-time and planned team, and the integrated team is the foundation.” The establishment of a full-time EMT requires continuous evaluation and verification. Firstly, establishing full-time EMTs conforms to the advanced emergency management concept and experience of developed countries. International experience has shown that establishing emergency relief organizations from top to bottom, constructing a national emergency management system, and establishing a series of full-time EMTs with efficient management procedures can significantly strengthen disaster response abilities. For example, the core concept of emergency management in the United States is “professional emergency response.” EMTs are required to have comprehensive emergency response capabilities to deal with various emergencies properly. All kinds of emergency positions are required to be held by personnel with professional titles, and emphasis is placed on emergency management training (43). The Singapore Civil Defense Force (SCDF), part of the National Civil Defense Scheme, specializes in civil defense and readiness tasks and has 215 emergency medical professionals (34). Secondly, full-time EMTs can offer several advantages in practice. They operate under an integrated management system and follow a standardized management operation mechanism, which can enhance overall mobilization, organization efficiency, logistical ease, and smooth transition. For example, the U.S. DMATs can be fully deployed within 8 h and are self-sustaining, equipped with food, water purifiers, generators, tents, and medicine for 72 h. Using standardized equipment and facilities, they can treat up to 250 patients per day (48).

## Limitations

The study has a few limitations. First, the investigation of this study only included the EMTs in North China (Tianjin). It would be more representative if samples from EMTs in more provinces and cities in China were included. In addition, we explored the characteristics of EMTs in North China from an overall perspective, individual perspective of different types of EMTs is lacking. Future research should focus on different types of EMTs, such as those dealing with chemical poisoning and nuclear radiation, to gain a more comprehensive understanding.

## Conclusion

Firstly, “bricolage” behavior can be used to define the current formation mode of EMTs in China. Due to limitations in human, material, and financial resources, EMTs' construction heavily relies on medical institutions and government funding. Secondly, the results of grounded theory revealed several drawbacks in the bricolage EMTs in North China, including chaotic management structure, weak internal stability, and incompetent support capability. To address these issues and achieve effective emergency medical rescue, it is necessary to establish a standardized personnel admission mechanism, full-time training and drill mechanism, diversified incentive mechanism, and multi-agent cooperation mechanism. Finally, the personnel admission process, incentive and assessment instrument, and resource support for EMTs hold great significance for developing countries in their efforts to construct effective EMTs.

## Data availability statement

The raw data supporting the conclusions of this article will be made available by the authors, without undue reservation.

## Ethics statement

The studies involving humans were approved by Ethics Committee of Tianjin Medical University (TMUhMEC2020024). The studies were conducted in accordance with the local legislation and institutional requirements. The participants provided their written informed consent to participate in this study.

## Author contributions

LW: Conceptualization, Data curation, Formal analysis, Investigation, Methodology, Project administration, Resources, Software, Supervision, Validation, Visualization, Writing – original draft, Writing – review & editing. Y-WS: Formal analysis, Investigation, Software, Writing – original draft, Writing – review & editing, Data curation, Methodology. X-YQi: Conceptualization, Project administration, Supervision, Validation, Writing – review & editing, Data curation. F-sL: Data curation, Methodology, Writing – review & editing. X-YQiu: Data curation, Methodology, Writing – review & editing. SS: Data curation, Methodology, Writing – review & editing. YD: Conceptualization, Funding acquisition, Project administration, Supervision, Validation, Writing – original draft, Writing – review & editing, Resources.
