# Supplementary material for: The bricolage mode of emergency medical teams in China: deficient and in urgent need of transformation—A qualitative study
Source: Front Public Health. 2024 Feb 16;12:1333820. doi: 10.3389/fpubh.2024.1333820 (PMC10904540; doi:10.3389/fpubh.2024.1333820)
Supplement: Supplementary file 1 [file Presentation_1.pdf]

Appendix 1: Interview outline
Appendix 2: The questionnaire of basic information of EMTs
Appendix 3: Basic information of EMTs of this study
Appendix 4: Training and drill of EMTs of this study
Appendix 5： Forty national EMTs in China

Appendix 1 Interview outline

1. When was your emergency medical rescue team or the medical team involved in rescue work established? How many rescuers are there? Is there recurrent financial support?

2.Do you think the existing resources (human, financial and material resources) of the emergency medical rescue team are sufficient or insufficient? If these resource are fully utilized, can they meet the existing work requirement?

3.In terms of material, personnel, technology, and system, how does your team usually integrate these elements? (e.g., Human resources: temporary team/permanent establishment team; Supplies: Spare equipment and timely procurement; System: update existing plans/make plans temporarily; Skills: Each team member has different skills/needs to learn new skills.)

4.①Based on the existing work, how about the quality of your rescue through this way of resource integration?

②Do you agree to use this resource bricolage method when participating in rescue work in the future?

③Regarding resource bricolage, what are the disadvantages of this approach? (e.g., waste of resources, the utilization of material reserves, team members do not adapt to work, etc.)

5.Do you often pay attention to all kinds of information or actively seek opportunities to improve the ability of the emergency medical rescue team?

6.What characteristics do you think team members/captains need to have to integrate and utilize the existing resources of the emergency medical rescue team? (e.g., creativity, integration of internal resources, social communication, and coordination ability, etc.)

7.What factors do you think limit the development of emergency medical teams? (e.g., resource constraints, part-time team, low team cohesion, less training and drills, lack of incentives etc.)

8.What is the way to break through these constraints? (e.g., resource support/personnel training, motivation/team atmosphere/collaboration, etc.)

Appendix 2 the questionnaire of basic information of EMTs

1. Team Name

____

2. Team level [Single choice]

□ International

□ National

□ Provincial

3. Team category [Single choice]

□ Emergency medical rescue

□ Chemical poisoning

□ Psychological crisis intervention

□ Nuclear radiation

□ Infectious disease

4.Team size

____

5. Professional composition of team personnel (e.g., logistics support, medical professionals and drivers)

____

6. Annual working funds

____

7. Source of funds [Multiple choice]

□ State financial support

□ Unit supply

□ Social Donations

□ Self-financing

□ Others __________________

8. Purpose of funds [Multiple choice]

□ Equipment support (e.g., tents, vehicles and other medical equipment)

□ Material support (e.g., protective equipment and medicines)

□ Guarantee funds for training and exercise

□ Personnel protection (e.g., grants, performance and allowances)

□ Others _________________

9. Training times (within the last year)

____

10. Training Method [Multiple choice]

□ Online course teaching and professional guidance

□ On-site teaching

□ Continuing Education

11. Training content [Multiple choice]

□ Stress training

□ Knowledge of medical emergency treatment

□ Skill training

□ Theoretical knowledge training

□ Emergency command training

□ Emergency plan preparation and application training

□ Others _________________

12. Number of drills (within the last year)

____

13. Exercise method [Multiple choice]

□ Long-distance training

□ Desktop drill

□ Simulation drill

Appendix 3 Basic information of EMTs of this study

Serial number	Team name/ Subsidiary units	Category of teams	Level of team	Number of team	Annual working expenses	Source of funds	Purpose of funds
1	Tianjin People's Hospital（Subsidiary units）	Comprehensive rescue	International& National	80	3 million	Government support	Equipment<br>Goods and materials<br>Training exercises<br>Security of personnel
2	Armed Police Specialty Medical Center（Subsidiary units）	Comprehensive rescue	National	100	1 million	Government support	Equipment<br>Goods and materials<br>Training exercises<br>Security of personnel
3	Tianjin Fourth Central Hospital（Subsidiary units）	Comprehensive rescue	Provincial	40	20 thousand	Hospital self-sufficiency	Goods and materials<br>Training exercises
4	Tianjin Nuclear and Radiological Emergency Response Team	Nuclear radiatio n treatment	Provincial	30	None	None	Other
5	Tianjin Anding Hospital（Subsidiary units）	Comprehensive rescue	Provincial	20	100 thousand	Government support	Equipment<br>Goods and materials<br>Training exercises
6	Tianjin Disease Control and Prevention Center（Subsidiary units）	Comprehensive rescue	Provincial	100	3.5 million	Government support	Goods and materials<br>Training exercises
7	Tianjin Occupational Disease Prevention Hospital chemical poisoning emergency bac kup rescue team	Poisoning treatment	Provincial	82	500 thousand	Government support	Goods and materials

Note: The subsidiary unit is mainly the supporting unit of the EMT, and when it is necessary to dispatch rescue, personnel selected by the hospital will form a team for rescue. EMT personnel are part-time.

Appendix 4 Training and drill of EMTs of this study

Serial number	Number of training	Training Methods	Content of Training	Number Of Drills	Drill Method
1	Three times in two years	Online course teaching and business guidance<br>On-site teaching (training course)<br>Continuing Education	Knowledge of medical emergency treatment Skills training<br>Theoretical knowledge<br>Emergency command capacity<br>Preparation and application of plan	Three times	Remote pulling<br>Desktop drill<br>Simulation drill
2	Two times	On-site teaching (training course)	Knowledge of medical emergency treatment<br>Skills training<br>Theoretical knowledge<br>Emergency command capacity	One time	Desktop drill
3	One time	On-site teaching (training course)	Knowledge of medical emergency treatment<br>Skills training	One time	Remote pulling
4	None	Online course teaching and business guidance	Other	None	Simulation drill
5	Three times	Online course teaching and business guidance	Skills training<br>Theoretical knowledge	Two times	Remote pulling<br>Desktop drill
6	Thirty times	On-site teaching (training course)<br>Continuing Education	Skills training<br>Theoretical knowledge	One time	Simulation drill
7	None	On-site teaching (training course)	Other	None	Simulation drill

Appendix 5 Forty national EMTs in China

No	Location	Team Name	Founding Institution/Organization	Year Established	References
1	Beijing	National Emergency Medical Rescue Team (Beijing)	Third Medical Center of PLA General Hospital	2001	http://qikan.cqvip.com/Qikan/Article/Detail?id=27837737
2	Beijing	National nuclear and biological rescue team	Beijing Military Region	2009	https://www.fx361.com/page/2015/0910/9315881.shtml
3	Jiangsu	Jiangsu National Nuclear and Radiation Emergency Health Emergency Team	Jiangsu Provincial Center for Disease Control and Prevention, Jiangsu Provincial People's Hospital, The Second Affiliated Hospital of Soochow University, the First People's Hospital of Lianyungang City, Jiangsu Provincial Institute of Health Supervision	2010	http://ccad.jiangsu.gov.cn/art/2017/7/7/art_214_6667247.html
4	Beijing	National Emergency Medical Rescue Team (Beijing)	China-Japan Friendship Hospital	2010	https://baijiahao.baidu.com/s?id=1677277457515637339&wfr=spider&for=pc
5	Tianjin	China International Emergency Medical Team and National Emergency Medical Rescue Team (Tianjin)	Tianjin Union Medical Center	2010	http://health.china.com.cn/2019-05/07/content_40741914.htm
6	Shanghai	National Emergency Medical Rescue Team (Shanghai)	Huashan Hospital Pudong Fudan University	2011	https://www.huashan.org.cn/news/detail/11196.html
7	Guangdong	China International Emergency Medical Team and National Emergency Medical Rescue Team (Guangdong)	Guangdong Second Provincial General Hospital	2011	https://www.cn-healthcare.com/articlewm/20170510/content-1014378.html?appfrom=jkj
8	Tianjin	National Emergency Medical Rescue Team (Tianjin)	Characteristic Medical Center of PAP	2011	https://kns.cnki.net/kcms2/article/abstract?v=3uoqIhG8C44YLTlOAiTRKibYlV5Vjs7ijP0rjQD-AVm8oHBO0FTadvNKK5VTq82vERGjEJreSkYayC4TJ9_7WOC1sJI3ufF3&uniplatform=NZKPT
9	Zhejiang	National Emergency Medical Rescue Team (Zhejiang)	Zhejiang Provincial People's Hospital	2011	http://www.hangzhou.gov.cn/art/2020/3/19/art_812270_42324490.html
10	Shanghai	China International Emergency Medical Team and National Emergency Medical Rescue Team (Shanghai)	Shanghai East Hospital	2012	http://www.scio.gov.cn/m/32621/32629/32755/Document/1484091/1484091.htm
11	Liaoning	National Emergency Medical Rescue Team (Liaoning)	The First hospital of China Medical University	2012	https://news.ifeng.com/c/7fcAqvu0823
12	Sichuan	National Emergency Medical Rescue Team and (Sichuan)	Sichuan Provincial People's Hospital	2012	https://www.samsph.com/new_media/2013/QbYQpMbz.html

13	Hunan	National Emergency Medical Rescue Team (Hunan)	The Second Xiangya Hospital of Central South University	2012	https://zyy.yilianmeiti.com/article/139288.html
14	Shaanxi	National Emergency Medical Rescue Team (Shaanxi)	Shaanxi Provincial People's Hospital	2012	https://news.sina.com.cn/o/2012-05-25/055924476237.shtml
15	Henan	National Emergency Medical Rescue Team (Henan)	The First Affiliated Hospital of Zhengzhou University	2012	http://www.scio.gov.cn/xwfbh/gssxwfbh/xwfbh/hubei/Document/1674460/1674460.htm
16	Shanxi	National Emergency Medical Rescue Team (Shanxi)	Shanxi Bethune Hospital/ Shanxi Dayi Hospital	2012	http://www.scio.gov.cn/xwfbh/gssxwfbh/xwfbh/hubei/Document/1674460/1674460.htm
17	Chongqing	National (Chongqing) emergency poisoning team	Chongqing Occupational Disease Prevention and Control Hospital (Municipal six Hospitals)	2012	https://baijiahao.baidu.com/s?id=1586209230514238501&wfr=spider&for=pc
18	Beijing	China Disease Control Emergency poisoning team	National Institute of Occupational Health and Poison Control, Chinese Center for Disease Control and Prevention	2012	http://www.oher.com.cn/fileZYWSYYJJY/journal/article/zywsyyjjy/2023/1/PDF/zywsyjjy-41-1-52.pdf
19	Beijing	Beijing Disease control emergency poisoning disposal team	Beijing Center for Disease Control and Prevention	2012	http://www.oher.com.cn/fileZYWSYYJJY/journal/article/zywsyyjjy/2023/1/PDF/zywsyjjy-41-1-52.pdf
20	Chongqing	National Emergency Medical Rescue Team (Chongqing)	The First Affiliated Hospital of Chongqing Medical University	2013	https://news.cqmu.edu.cn/info/1003/9019.htm
21	Xinjiang	National Emergency Medical Rescue Team (Xinjiang)	People's Hospital of Xinjiang Uygur Autonomous Region	2013	https://baike.baidu.com/item/%E6%96%B0%E7%96%86%E5%9B%BD%E5%AE%B6%E7%B4%A7%E6%80%A5%E5%8C%BB%E5%AD%A6%E6%95%91%E6%8F%B4%E9%98%9F/8937732
22	Yunnan	National Emergency Medical Rescue Team (Yunnan)	First People's Hospital of Yunnan Province	2014	https://new.qq.com/rain/a/20220622A0CCDR00
23	Guangxi	National Emergency Medical Rescue Team (Guangxi)	Beihai People′s Hospital	2015	http://news.gxnews.com.cn/staticpages/20150205/newgx54d2b07f-12173349.shtml
24	Jilin	National Emergency Medical Rescue Team (Jilin)	The First Bethune Hospital of Jilin University	2016	https://www.jdyy.cn/index.php?m=home&c=View&a=index&aid=56868
25	Guangdong	National Mobile Health Emergency Response Center for Nuclear Radiation (Guangdong)	Guangdong Occupational Disease Prevention Hospital	2016	https://baijiahao.baidu.com/s?id=1586209230514238501&wfr=spider&for=pc
26	Fujian	National Emergency Medical Rescue Team (Fujian)	Fujian Provincial Hospital	2017	https://news.fznews.com.cn/shehui/20170611/593c890691635.shtml
27	Guizhou	National Health Emergency Mobile Treatment Center for Poisoning Incidents (Guizhou)	Guizhou Provincial People's Hospital, Guizhou Provincial Center for Disease Control and	2017	https://www.gz5055.com/news_kd/2022/kazxP5dJ.html

			Prevention, the Third People's Hospital of Guizhou Province		
28	Jiangxi	National Emergency Medical Rescue Team (Jiangxi)	Jiangxi Provincial People's Hospital	2017	http://www.jiangxi.gov.cn/art/2017/11/12/art_393_214313.html?xxgkhide=1
29	Guizhou	National Emergency Medical Rescue Team (Guizhou)	The Affiliated Hospital of Guizhou Medical University	2017	https://www.gmc.edu.cn/info/1059/10941.htm
30	Sichuan	China International Emergency Medical Team and National Emergency Medical Rescue Team (Sichuan)	West China Hospital of Sichuan University	2018	http://www.cd120.com/public/about/emergency/procedures/33270.html
31	Jiangsu	National Emergency Medical Rescue Team (Jiangsu)	Jiangsu Province Hospital	2018	http://www.jiangsu.gov.cn/art/2018/6/22/art_46501_7690343.html
32	Anhui	National Health Emergency Mobile Medical Treatment Center (Anhui) Emergency Medical Rescue Team	The First Affiliated Hospital of Anhui Medical University	2018	https://wjw.ah.gov.cn/public/7001/52087191.html
33	Beijing	Chinese rescue team	Beijing Fire Brigade, Emergency General Hospital, China Earthquake Emergency Search and Rescue Center	2018	https://www.mtzyy.com.cn/Html/Mobile/Articles/1026.html
34	Hainan	National Emergency Medical Rescue Team (Hainan)	Hainan General Hospital, The First Affiliated Hospital of Hainan Medical University, The Second Affiliated Hospital of Hainan Medical University	2019	https://www.hainmc.edu.cn/ecmd/info/1351/3634.htm
35	Xinjiang Production and Construction Corps	National Emergency Medical Rescue Team (Xinjiang Production and Construction Corps)	The First Affiliated Hospital of Shihezi University	2019	http://wx.ihwrm.com/baokan/article/info.html?doc_id=3418546
36	Macau	China International Emergency Medical Team (Macau)	Health Bureau of the Macao Special Administrative Region Government	2019	http://dwq.people.cn/n2/2019/0505/c386999-32905052.html
37	Ningxia	National Emergency Medical Rescue Team (Ningxia)	General Hospital of Ningxia Medical University	2020	http://www.gov.cn/xinwen/2020-01/06/content_5466892.htm?_zbs_baidu_bk
38	Shandong	National Health Emergency Mobile Treatment Center for Poisoning Incidents (Shandong)	Shandong Provincial Hospital, Shandong Institute of Occupational Health and Occupational Disease Prevention	2020	https://baijiahao.baidu.com/s?id=1683595808256845628&wfr=spider&for=pc
39	Hebei	National Health Emergency Mobile Medical Treatment Center (Hebei)	The Third Hospital of Hebei Medical University	2020	https://www.163.com/dy/article/FL81Q8MV0514IJUD.html

Note:National Health Commission of China. Transcript of press conference under the Joint Prevention and Control Mechanism of The State Council on February 27,

2023. http://www.nhc.gov.cn/xcs/yqfkdt/202302/87dc9bd631cf401fb9132c4a7e502e1c.shtml (Acce-ssed May 11, 2023) The 40th Chinese national EMT was not fou
nd
